# Interim estimates of the effectiveness of influenza vaccination against influenza‐associated hospitalization in children in Hong Kong, 2015–16

**DOI:** 10.1111/irv.12399

**Published:** 2016-10-14

**Authors:** Benjamin J. Cowling, Mike Y. W. Kwan, Joshua S. C. Wong, Shuo Feng, Chi‐Wai Leung, Eunice L. Y. Chan, Kwok‐Hung Chan, Tak‐Keung Ng, Wing‐Kin To, Malik J. S. Peiris, Susan S. Chiu

**Affiliations:** ^1^ WHO Collaborating Centre for Infectious Disease Epidemiology and ControlSchool of Public HealthLi Ka Shing Faculty of MedicineThe University of Hong KongHong Kong Special Administrative RegionChina; ^2^ Department of Paediatrics and Adolescent MedicinePrincess Margaret HospitalHong Kong Special Administrative RegionChina; ^3^ Department of Paediatrics and Adolescent MedicineQueen Mary Hospital and Li Ka Shing Faculty of MedicineThe University of Hong KongHong Kong Special Administrative RegionChina; ^4^ Department of MicrobiologyLi Ka Shing Faculty of MedicineThe University of Hong KongHong Kong Special Administrative RegionChina; ^5^ Department of PathologyPrincess Margaret HospitalHong Kong Special Administrative RegionChina; ^6^ Department of PathologyYan Chai HospitalHong Kong Special Administrative RegionChina; ^7^ Center of Influenza ResearchLi Ka Shing Faculty of MedicineThe University of Hong KongHong Kong Special Administrative RegionChina

**Keywords:** effectiveness, hospitalization, influenza, interim, pediatrics, vaccine

## Abstract

From 1 September 2015 through 31 January 2016, we enrolled 2068 children 6 months to 17 years of age admitted to hospital with a febrile acute respiratory infection in our test‐negative study. Information on receipt of 2015–16 northern hemisphere inactivated influenza vaccination was elicited from parents or legal guardians. Using conditional logistic regression adjusting for age and matching on calendar time, we estimated influenza vaccine effectiveness against hospitalization with influenza A or B to be 79.2% (95% confidence interval: 42.0%–92.4%). Annual influenza vaccination should be more widely used in children in Hong Kong.

## 1 Introduction

Influenza viruses circulate each year, causing a considerable burden to public health. Children typically face the greatest risk of influenza virus infection among all age groups in a population,^1^ and young children have high rates of hospitalizations associated with influenza.^2^, ^3^ Influenza vaccination is the most effective measure to prevent infection and recommended by the World Health Organization for many high‐risk groups including young children from 6 to 59 months of age.^4^ In Hong Kong, a subtropical city on the south coast of China, all children between 6 months and 6 years of age and children 6 years of age or above with an underlying condition associated with an increased risk for influenza complications are recommended to receive influenza vaccination each year.^5^ The Hong Kong government provides a subsidy for influenza vaccination for children between 6 months and 6 years of age and older children from low‐income families, while children between 6 months and 17 years of age with an underlying medical condition can receive free influenza vaccination.^6^

It is informative to evaluate influenza vaccination effectiveness (VE) each season to confirm that vaccination is providing adequate protection, to inform risk communication and to provide evidence for vaccination strategies and other public health measures.^7^ Timely interim, or mid‐season, estimates of influenza VE can be particularly useful to guide resource allocation or implement additional preventive measures if VE is low.^8^

## 2 Methods

### 2.1 Study design

We used the test‐negative study design, which is a type of case–control design, to estimate influenza VE.^6^, ^7^, ^9^, ^10^, ^11^ We enrolled children admitted to Queen Mary Hospital 1 of 2 public hospitals with inpatient paediatric services on Hong Kong Island, and the Princess Margaret Hospital and Yan Chai Hospital network which are 2 of the 3 public hospitals in the West Kowloon district with inpatient paediatric services. The same study protocol was used in each hospital. Children 6 months to 17 years of age admitted to the general wards of these hospitals with a febrile acute respiratory infection, defined as fever measured ≥38°C with any respiratory symptom such as cough, runny nose or sore throat, were eligible for inclusion in this study. Children with risk factors for potentially severe respiratory infections such as prematurity or chronic lung disease were not excluded. Nasopharyngeal aspirates were obtained from all patients and tested for influenza A and B virus by direct immunofluorescence assay (for rapid diagnosis) followed by reverse transcriptase polymerase chain reaction for seasonal influenza A and B viruses using laboratory methods as previously described.^6^, ^11^

Influenza vaccination history within 6 months of hospitalization was elicited from the parents or legal guardians of patients using a standardized questionnaire administered by research personnel. Vaccinated children were those who had received influenza vaccination within the 6 months prior to admission in a regimen and dosage appropriate for age and influenza vaccination history according to the recommendations of the Advisory Committee on Immunization Practices, with the last dose more than 2 weeks before hospitalization.^12^ Children who should receive 2 doses of influenza vaccination, but only received 1 dose or were vaccinated within 2 weeks of hospitalization were categorized as unvaccinated. The northern hemisphere formulation of trivalent and quadrivalent inactivated influenza vaccines were used during our study period. Analysis was performed by R 3.1.1.

### 2.2 Ethical approval

The study protocol was approved by the Institutional Review Board of the University of Hong Kong and that of the Kowloon West Cluster Research Ethics Committee which waived the need for written consent as viral investigation was a routine diagnostic test carried out as part of routine care, any patient information was delinked from individual patient identification to maintain patient confidentiality and participation by responding to the questionnaire was voluntary and indicative of consent.

### 2.3 Statistical analysis

Following the analytic approach used in previous years,^6^, ^11^ we used conditional logistic regression models for influenza status versus vaccination status, adjusting for age and age squared, and matching on calendar time. We matched for calendar time because influenza vaccine coverage changes over time during prolonged periods of influenza activity in Hong Kong.^6^, ^11^ VE was estimated as one minus the adjusted conditional odds ratio, multiplied by 100%. Statistical analyses were performed in R version 3.1.1 (R Foundation for Statistical Computing, Vienna, Austria).

## 3 Results

**Figure 1:**
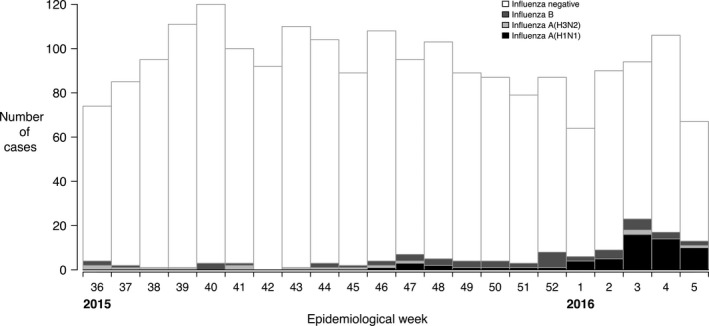
Enrolment of children from 1 September 2015 through 31 January 2016 in Hong Kong, classified by laboratory testing result for influenza by type/subtype

We enrolled 2068 children from 1 September 2015 through 31 January 2016. A small number of children with unknown vaccination history (n = 8) or unknown laboratory results (n = 11) were excluded, leaving data on 2049 children for inclusion in analyses. Of the 2049 children, 59 (2.9%) tested positive for influenza A(H1N1), 15 (0.7%) tested positive for influenza A(H3N2), 49 (2.4%) tested positive for influenza B and the remainder tested negative for influenza. The characteristics of enrolled patients are shown in Table 1. Patients testing negative for influenza were enrolled at fairly constant rates throughout the period, while most patients testing positive for influenza were enrolled in January 2016, with the early part of the influenza season being dominated by A(H1N1) (Fig. 1).

**Table 1:** Comparison of cases testing positive for influenza A and B and test‐negative cases in Hong Kong, 2015–16

Characteristic	Test‐positive for influenza A or B(***n*** = 123)N (%)	Test‐negative for influenza A and B(***n*** = 1926) N (%)	*P*‐value^a^
Age group			
6 mo–2 y	40 (32.5%)	980 (50.9%)	<0.01
3–5 y	39 (31.7%)	570 (29.6%)	
6–17 y	44 (35.8%)	376 (19.5%)	
Male	77 (62.6%)	1068 (55.5%)	0.15
Receipt of TIV/QIV^b^ in the preceding 6 mo	4/123 (3.3%)	279/1926 (14.5%)	<0.01
Breakdown of overall vaccination coverage by age			
6 mo–2 y	0/40 (0%)	87/980 (8.9%)	0.09
3–5 y	1/39 (2.6%)	135/570 (23.7%)	<0.01
6–17 y	3/44 (6.8%)	57/346 (15.2%)	0.21
Breakdown of vaccines received by vaccine type:			
Vaccine type: TIV^b^	3/4 (75.0%)	127/279 (45.5%)	0.34
Vaccine type: QIV^b^	0/4 (0%)	65/279 (23.3%)	0.58
Vaccine type: unknown	1/4 (25.0%)	87/279 (31.2%)	1.00

a *P*‐values estimated by chi‐squared tests and Fisher's exact test.

b TIV, trivalent inactivated influenza vaccine, QIV, quadrivalent inactivated influenza vaccine.

**Figure 2:**
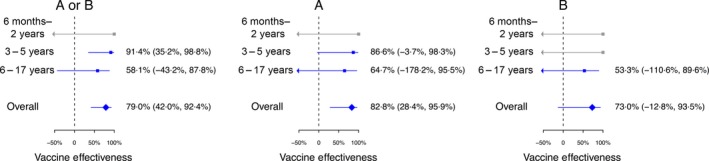
Interim estimates of influenza vaccine effectiveness against influenza viruses overall and by influenza type and age group in Hong Kong, 2015–16. Vaccination effectiveness estimates of 100% with wide confidence intervals are from subgroups in which there were no vaccinated children in the test‐positive group and are shown in grey

We estimated influenza VE to be 79.0% (95% confidence interval, CI: 42.0, 92.4) against any influenza, 82.8% (95% CI: 28.4, 95.9) against influenza A and 73.0% (95% CI: −12.8, 93.5) against influenza B among children aged 6 months to 17 years. Age‐specific estimates of VE overall and by influenza type are shown in Fig. 2. We did not have sufficient data to estimate VE against influenza A subtypes.

## 4 Discussion

Influenza vaccination coverage was only 14% in the hospitalized children despite government subsidies for younger children and children at higher risk of complicated infection. Our preliminary estimates of influenza VE for the 2015–16 northern hemisphere influenza vaccine were at the high end of VE against influenza hospitalization compared to estimates in the same age group from previous years in Hong Kong^6^, ^11^: VE against overall influenza A or B hospitalization was 61.7% (95% CI: 43.0, 74.2) in 2009–2013 and against influenza B hospitalization was 47.6% (95% CI: 10.0, 69.4) in 2009–2014.. Studies conducted in outpatient settings for all ages reported modest to high early/interim VE estimates: Canada recently reported VE of 64% (95% CI: 44–77) against influenza A(H1N1)^13^; the I‐MOVE study reported VE of 46.3% (4.9–69.7%) against any influenza^14^; the United States reported an interim estimate of VE of 59%^15^; and the United Kingdom reported an interim estimate of 41.5% against any influenza.^16^

There are some limitations of our study. We estimated VE against hospitalization, and this will differ from VE against infection or medically attended infection if vaccination modifies disease severity.^10^ In a recent review, estimates of VE against hospitalization were very similar to estimates of VE against medically attended illness, for studies conducted in the same populations, years and age groups.^17^ Influenza vaccination history of 6 months was self‐reported, and misclassification of vaccination status may have biased estimates of VE.^18^ Information was not available on potential confounders such as underlying medical conditions. The sample size was large enough to confirm statistically significant overall VE, but not sufficient to provide precise estimates of VE in some age groups. Finally, our interim estimates of VE may differ from the future end‐of‐season estimate, although usually interim estimates are quite similar to final end‐of‐season estimates in test‐negative studies.^8^, ^19^, ^20^ Final estimates for 2015–16 in Hong Kong will be available around the end of 2016.

In conclusion, we documented that influenza vaccination was associated with good protection against influenza‐associated hospitalization in children 6 months to 17 years in Hong Kong in this interim analysis. Healthcare providers and parents should be encouraged to increase influenza vaccination coverage in children in Hong Kong.

## Potential Conflict of Interests

BJC has received research funding from MedImmune Inc and Sanofi Pasteur, and consulted for Crucell NV. JSMP has received research funding from Crucell NV and serves as an *ad hoc* consultant for GlaxoSmithKline and Sanofi. The authors report no other potential conflict of interests.

## Author Contributions

SSC conceived the study. MYWK, JSCW and ELYC collected data. BJC, SF and ELYC analysed the data. All authors interpreted the data. BJC wrote the first draft and all authors contributed to review and revision and have seen and approved the final version.

## References

[1] Monto AS , Koopman JS , Longini IM, Jr . Tecumseh study of illness. XIII. Influenza infection and disease, 1976–1981. Am J Epidemiol. 1985;121:811–822. 4014174 10.1093/oxfordjournals.aje.a114052

[2] Zhou H , Thompson WW , Viboud CG , et al. Hospitalizations associated with influenza and respiratory syncytial virus in the United States, 1993–2008. Clin Infect Dis. 2012;54:1427–1436. 22495079 10.1093/cid/cis211 PMC3334364

[3] Chiu SS , Lo JY , Chan KH , et al. Population‐based hospitalization burden of influenza a virus subtypes and antigenic drift variants in children in Hong Kong (2004–2011). PLoS One. 2014;9:e92914. 24786780 10.1371/journal.pone.0092914 PMC4005733

[4] World Health Organization . Meeting of the Strategic Advisory Group of Experts on immunization, April 2012–conclusions and recommendations. Wkly Epidemiol Rec. 2012;87:201–216. 24340402

[5] Center for Health Protection . Protect your child from seasonal influenza. 2013.

[6] Cowling BJ , Chan KH , Feng S , et al. The effectiveness of influenza vaccination in preventing hospitalizations in children in Hong Kong, 2009–2013. Vaccine. 2014;32:5278–5284. 25092636 10.1016/j.vaccine.2014.07.084 PMC4165553

[7] Jackson ML , Nelson JC . The test‐negative design for estimating influenza vaccine effectiveness. Vaccine. 2013;31:2165–2168. 23499601 10.1016/j.vaccine.2013.02.053

[8] Leung VK , Cowling BJ , Sullivan SG . Concordance of interim and final estimates of influenza vaccine effectiveness: a systematic review. Euro Surveill. 2016;21. doi: 10.2807/1560‐7917.ES.2016.21.16.30202. 10.2807/1560-7917.ES.2016.21.16.30202 27124573

[9] Kelly H , Carville K , Grant K , Jacoby P , Tran T , Barr I . Estimation of influenza vaccine effectiveness from routine surveillance data. PLoS One. 2009;4:e5079. 19333374 10.1371/journal.pone.0005079 PMC2658741

[10] Foppa IM , Ferdinands JM , Chaves SS , et al. The case test‐negative design for studies of the effectiveness of influenza vaccine in inpatient settings. Int J Epidemiol. 2016; pii: dyw022. [Epub ahead of print]. 10.1093/ije/dyw022 PMC5025336 26979985

[11] Chiu SS , Feng S , Chan KH , et al. Hospital‐based vaccine effectiveness against influenza B lineages, Hong Kong, 2009–14. Vaccine. 2016;34:2164–2169. 27013437 10.1016/j.vaccine.2016.03.032

[12] Grohskopf LA , Sokolow LZ , Olsen SJ , Bresee JS , Broder KR , Karron RA . Prevention and control of influenza with vaccines: recommendations of the advisory committee on immunization practices, United States, 2015–16 Influenza Season. MMWR Morb Mortal Wkly Rep. 2015;64:818–825. 26247435 10.15585/mmwr.mm6430a3 PMC5779578

[13] Chambers C , Skowronski DM , Sabaiduc S , et al. Interim estimates of 2015/16 vaccine effectiveness against influenza A(H1N1)pdm09, Canada, February 2016. Euro Surveill. 2016;21: doi: 10.2807/1560‐7917.ES.2016.21.11.30168. 10.2807/1560-7917.ES.2016.21.11.30168 27020673

[14] Kissling E , Valenciano M . Early influenza vaccine effectiveness results 2015–16: I‐MOVE multicentre case‐control study. Euro Surveill. 2016;21: doi: 10.2807/1560‐7917.ES.2016.21.6.30134. 10.2807/1560-7917.ES.2016.21.6.30134 26898240

[15] Centers for Disease Control and Prevention . Flu Vaccine Nearly 60 Percent Effective. 2016.

[16] Pebody R , Warburton F , Ellis J , et al. Effectiveness of seasonal influenza vaccine in preventing laboratory‐confirmed influenza in primary care in the United Kingdom: 2015/16 mid‐season results. Euro Surveill. 2016;21: doi: 10.2807/1560‐7917.ES.2016.21.13.30179. 10.2807/1560-7917.ES.2016.21.13.30179 27074651

[17] Feng S , Cowling BJ , Sullivan SG . Influenza vaccine effectiveness by test‐negative design ‐ Comparison of inpatient and outpatient settings. Vaccine. 2016;34:1672–1679. 26920469 10.1016/j.vaccine.2016.02.039 PMC4826670

[18] Sullivan SG , Feng S , Cowling BJ . Potential of the test‐negative design for measuring influenza vaccine effectiveness: a systematic review. Expert Rev Vaccines. 2014;13:1571–1591. 25348015 10.1586/14760584.2014.966695 PMC4277796

[19] Jimenez‐Jorge S , Pozo F , Larrauri A , cycEVA Study Team . Interim influenza vaccine effectiveness: a good proxy for final estimates in Spain in the seasons 2010–2014. Vaccine. 2015;33:3276–3280. 25869892 10.1016/j.vaccine.2015.03.051

[20] Belongia EA , Kieke BA , Donahue JG , et al. Influenza vaccine effectiveness in Wisconsin during the 2007–08 season: comparison of interim and final results. Vaccine. 2011;29:6558–6563. 21767593 10.1016/j.vaccine.2011.07.002

